# Comparison of Activator-Headgear and Twin Block Treatment Approaches in Class II Division 1 Malocclusion

**DOI:** 10.1155/2017/4861924

**Published:** 2017-01-22

**Authors:** Stjepan Spalj, Kate Mroz Tranesen, Kari Birkeland, Visnja Katic, Andrej Pavlic, Vaska Vandevska-Radunovic

**Affiliations:** ^1^Department of Orthodontics, School of Medicine, University of Rijeka, Rijeka, Croatia; ^2^Private Practice, Tannregulering Kristiansand, Kristiansand, Norway; ^3^Department of Orthodontics, Institute of Clinical Dentistry, University of Oslo, Oslo, Norway

## Abstract

The purpose was to compare the treatment effects of functional appliances activator-headgear (AH) and Twin Block (TB) on skeletal, dental, and soft-tissue structures in class II division 1 malocclusion with normal growth changes in untreated subjects. The sample included 50 subjects (56% females) aged 8–13 years with class II division 1 malocclusion treated with either AH (*n* = 25) or TB (*n* = 25) appliances. Pre- and posttreatment lateral cephalograms were evaluated and compared to 50 untreated class II division 1 cases matched by age, gender, ANB angle, and skeletal maturity. A paired sample, independent samples tests and discriminant analysis were performed for intra- and intergroup analysis. Treatment with both appliances resulted in significant reduction of skeletal and soft-tissue facial convexity, the overjet, and the prominence of the upper lip in comparison to untreated individuals (*p* < 0.001). Retroclination of maxillary incisors and proclination of mandibular incisors were seen, the latter being significantly more evident in the TB group (*p* < 0.05). Increase of effective mandibular length was more pronounced in the TB group. In conclusion, both AH and TB appliances contributed successfully to the correction of class II division 1 malocclusion when compared to the untreated subjects with predominantly dentoalveolar changes.

## 1. Introduction

Early treatment of class II malocclusion aims to correct the sagittal relationship, modify the pattern of facial growth, and improve both hard- and soft-tissue profile [1–4]. The majority of the clinical studies recognize the useful effect of functional appliances in sagittal correction of the malocclusion but agree that the treatment is mainly restricted to dentoalveolar changes [5]. Favorable skeletal changes which can modify the growth pattern can also occur depending on individual growth potential [1, 6].

A class II malocclusion may result from mandibular deficiency, maxillary excess, or combination of both [7, 8]. Several varieties of functional appliances are currently in use aiming to correct the skeletal imbalances. The combination of an activator with headgear (AH) is used to provide greater cumulative skeletal changes than either appliance would provide alone [9]. They affect maxilla by decreasing forward and downward growth of the maxillary complex, while allowing the forward growth of the mandible to continue, thus influencing the profile more favorably [9, 10]. Twin Block (TB) appliance as well as most of other functional appliances is designed to encourage adaptive skeletal growth by maintaining the mandible in a corrected forward position for a sufficient period of time [1, 4, 11].

Many studies have investigated the effect of AH and TB appliance on the dental and skeletal variables. However, no studies have provided a direct comparison of the treatment changes between them. One study compared the effects of both appliances [3], but the evaluation was limited to the soft-tissue profile changes.

Therefore, the aims of this study were to explore skeletal and dentoalveolar changes in class II division 1 patients treated with TB and AH and to compare their treatment effect with normal growth changes of untreated controls (CTRL) with the same malocclusion. The hypotheses were as follows:

1. Both appliances have more pronounced dentoalveolar effect in the treated groups than growth itself in the untreated group.
2. TB stimulates more skeletal growth of mandible than AH.
3. AH has better control of vertical dimension than TB.

## 2. Materials and Methods

### 2.1. Study Population and Design

The sample included 50 subjects (56% females) aged 8–13 years (median 11) with class II division 1 malocclusion treated with either AH (*n* = 25) or TB (*n* = 25) appliances. The data were collected retrospectively among 151 subjects treated in the period of 2000–2015 at the Department of Orthodontics in Oslo, Norway, and the Department of Orthodontics in Rijeka, Croatia. Inclusion criteria were distal molar occlusion, overjet (OJ) >5 mm, and having pre- and posttreatment lateral cephalograms. According to the cervical vertebral maturation method [12], the included subjects were in the prepeak stages (CS1–CS3) of skeletal maturation before treatment and CS3–CS5 after treatment.

The AH appliance had all maxillary teeth covered with acrylic and included labial spring for the torque control of the incisors [13]. High pull headgear was always used simultaneously with the appliance. TB appliance [14] with addition of maxillary labial bow to aid the anterior retention and make the maxillary incisors retroclined was used in the other group. The expansion screw was incorporated in the maxillary plate and activated one quarter-turn each week for an average period of six months. Construction bite was the same in both appliances with the anterior positioning of mandible by 6 mm and vertical opening by 4 mm in the first molar area. The patients were recommended to use the appliances for 12–14 hours per day. Treatment was stopped when the patients achieved molar class I occlusion or slight hypercorrection.

**Figure 1:**
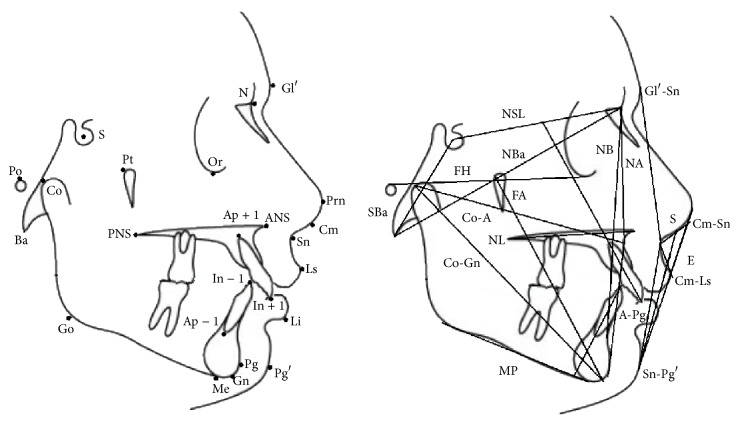
Points and plains used in cephalometric analysis: Gl′ (soft-tissue glabella), Prn (pronasale), Cm (columella), Sn (subnasale), Ls (labrale superius), Li (labrale inferius), Pg′ (soft-tissue pogonion), Pg (osseous pogonion), Gn (gnathion), Me (menton), Go (gonion), Ba (basion), Co (condylion), Po (porion), Pt (pterygoid point), S (sella), PNS (posterior nasal spine), ANS (anterior nasal spine), N (nasion), Or (orbitale), Ap + 1 (apicale superius), Ap − 1 (apicale inferius), In + 1 (incisale superius), In − 1 (incisale inferius), NSL (nasion-sella line), FA (facial axis), NL (nasal line), MP (mandibular plane), FH (Frankfort horizontal), E (Ricketts' Esthetic line), and S (Steiner's line).

Pretreatment (T1) and posttreatment (T2) lateral cephalograms were evaluated and compared to 50 untreated class II division 1 cases matched by age, gender, ANB angle, skeletal maturation of cervical vertebrae, and observation period. They were selected from American Association of Orthodontists Foundation Craniofacial Growth Legacy Collection. Cephalometric analysis (Table 1, Figure 1) was performed on calibrated pre- and posttreatment lateral cephalograms by two investigators (KMT and SS) using the cephalometric software Facad (Ilexis AB, Sweden) and AudaxCeph (Audax, Slovenia).

The study was in accordance with the Helsinki Declaration and the protocol was approved by the local ethical committees in Norway (02-09-2010) and in Croatia (2170-24-01-15-2).

### 2.2. Statistical Analysis

After inspection of histograms and quantile-quantile plots and testing the normality of the data with the Shapiro-Wilk test, a paired *t*-test was preformed to assess the statistical significance of changes occurring during the treatment with each appliance (intragroup analysis). Independent samples test was used for intergroup analysis (between appliances groups). For the differences in age between groups, the Kruskal-Wallis test was used and *χ*^2^ for differences in gender. Analysis of variance with the Student-Newman-Keuls post hoc test was used to test the amount of changes between treated groups and controls. Effect size, that is, the magnitude of the relationship, was estimated by *r* and *η*^2^. Discriminant function analysis, a multivariate technique, was used to explore which changes in cephalometric parameters discriminate treatment groups and untreated subjects the most, and how effective those parameters are in predicting treatment group membership.

Reliability, that is, consistency of measurements, was assessed on ten randomly selected cephalograms remeasured with a three-month interval. Intraclass correlation coefficient (ICC) and Dahlberg formula were used. Dahlberg formula for method error is ME = ∑*d*^2^/2*n*, where “*d*” is the difference between two registrations and “*n*” is the number of double registrations [15]. IBM SPSS 22 (IBM Corp, Armonk, USA) software was used for data analysis.

## 3. Results

The reliability of measurements was good or excellent, with ICC ranging from 0.660 for upper-to-lower facial height ratio to 0.995 for inclination of mandibular plane relative to the anterior cranial base and ME from 0.3 for SNA to 6.6 for nasolabial angle. The error of the method was less than 10% of the biologic variation. Power calculation of this study showed the least detectable mean difference in diff ANB to be 1.1 degree (80% test power with 95% significance level). The present study was not suitable for statistical analysis of gender differences in treatment effects due to small samples.

At T1, the treatment groups had similar characteristics (Tables 2 and 3), and differences between genders in those variables were not significant. Untreated subjects had lower OJ at T1 (5.6 ± 2.1; *p* < 0.001), but higher OJ at T2 (5.9 ± 2.2; *p* < 0.001) compared to the treated groups.

Treatment with both appliances resulted in significant increase of the SNB angle, reduction of the ANB angle, retrusion and retroclination of the maxillary incisors, protrusion and proclination of the mandibular incisors, and reduction of the OJ (Table 3). Soft tissues demonstrated reduction of convexity and prominence of the upper lip and increased nasolabial angle.

The untreated group manifested significant increase in the SNB angle (*p* = 0.005), upper and lower facial height (*p* ≤ 0.001), and increased maxillary and mandibular length (*p* < 0.001), with the mandible growing significantly more than the maxilla (Table 4).

Treatment with the TB appliance resulted in increased mandibular incisor proclination and protrusion compared to the AH appliance (*p* < 0.05; Table 5).

Treatment with both functional appliances resulted in significant reduction of the ANB angle when compared to the untreated population (*p* < 0.001; Table 5). It was mainly due to the increase in the SNB angle and maxillomandibular differential length (difference between effective mandibular length (Co-Gn) and the effective midface length (Co-A); *p* < 0.001). Both appliances significantly reduced the convexity of the hard and soft facial tissues in comparison to the untreated population (*p* < 0.001). Additionally, retroclination of the maxillary incisors was noticed in both treatment groups and was slightly but insignificantly more pronounced in the AH group. Proclination of the mandibular incisors was significantly more pronounced in the TB group (*p* < 0.05). As a consequence, OJ and the prominence of the upper lip were significantly reduced in comparison to the untreated subjects (*p* < 0.001).

**Figure 2:**
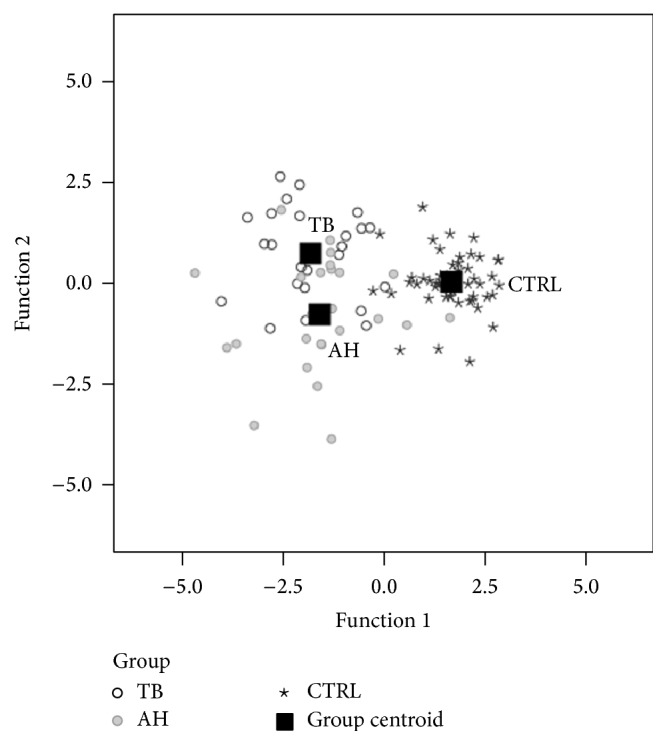
Canonical discriminant functions graph.

In order to explore which variables mostly distinguish the three groups of subjects, discriminant analysis was applied. Changes in cephalometric variables during treatment and observation period were used as predictors. Variables that demonstrated most changes or differences were selected, with special attention in obtaining the lowest possible correlation between predictors. Two discriminant functions in this analysis could be estimated, both having significant discriminating power.  Figure 2 demonstrates that functions clearly discriminate groups. First discriminant function, presented in horizontal direction of Figure 2, distinguishes treated from untreated subjects. Variables that comprise this first discriminant function are presented in Table 6 and their correlations with the first discriminant function are marked with asterisks in the first numeric column. More effect size was seen in the position of the incisors and soft tissues than in the skeletal changes. Changes in those features explained high proportion of variability of distinction between treated and untreated subjects (90.9%; *p* < 0.001).

Second discriminant function, presented in vertical direction of Figure 2 and marked in the last column of Table 6, mostly distinguishes the two treatment groups. More effect size was seen in inclination of incisors and mandibular growth than in the position of the lower lip. Changes in those features accounted for low variability of distinction between treatment groups (9.1%; *p* = 0.041). Discriminant analysis correctly classified 79% of the subjects. Correct group membership was retained in 96% untreated subjects, 72% of TB, and 52% of AH group.

## 4. Discussion

Both TB and AH functional appliances successfully reduced the severity of class II malocclusion by a combination of dental and skeletal changes. Overjet, SNB, and ANB angles were significantly improved in both groups. All of these changes were significantly different from the changes in the untreated, control group suggesting positive treatment effect with functional appliances. The only variables that exhibited significant differences between the two appliances after the treatment were the proclination and the protrusion of the mandibular incisors, which were more pronounced in the TB group.

The SNB angle significantly increased in both treatment groups, which is in agreement with other studies [8, 13, 16]. However, these changes, particularly in the TB group, were smaller than the previously reported and could be related to the concomitant increase in the lower anterior facial height, lower incisor proclination, and posterior displacement of point B [14].

Great variability in increase in effective mandibular length, that is, Co-Gn, is demonstrated, particularly in AH group. The effective mandibular length increased mostly in the TB group which is supported by numerous investigations [2, 5, 8]. The amount of mean increase in mandibular length in the AH group is similar to normal mandibular growth of untreated class II division 1 cases. Supplementary mandibular length growth of 2.5 mm in the TB subjects in comparison to untreated subjects in this study corresponds with the results reported in a recent meta-analysis [17]. One of the several systematic reviews on the treatment effect of removable functional appliances reported that short-term evidence suggested mainly dentoalveolar rather than skeletal effects; however, the skeletal changes were more pronounced with the TB appliance [5]. The most recent meta-analysis revealed more supplementary mandibular growth in pubertal than prepubertal class II malocclusion patients treated with functional appliances [18]. Therefore, treatment timing, as well as individual differences in treatment response, may give a plausible explanation for the reported discrepancies.

Both appliances in the current study had little, insignificant restraining effect on the maxilla. Several investigations have previously reported that forward growth of the maxilla may be inhibited during AH treatment [4, 9, 10, 16]. Others could not confirm this effect [13, 19]. Restricted forward growth of maxilla in patients treated with TB is found in most of the studies included in the systematic review by Ehsani et al. [17]. The labial bow used to increase retention and control the maxillary incisors in the TB appliance might have made the maxillary incisors retroclined, made the roots proclined, and affected the position of the A point [11]. Thus, it is possible that the restraining effect on maxilla was more pronounced but was underestimated due to a forward movement of the A point. The increased SNA angle in the control group is also in support of this notion.

The ANB angle showed higher decrease in both treatment groups in comparison to the untreated controls. The significant change in the TB group was mainly due to the significant skeletal mandibular effect concerning both angular and linear measurements. In the AH group, the nature of the ANB changes is controversial and could be a combination of dentoalveolar and skeletal changes in both jaws. Some studies indicate that reduction of the ANB angle is mainly due to a delayed forward growth of maxilla, while some report that reduced ANB angle is more dependent on increased mandibular growth [16]. Regardless of the treatment changes that lead to reduction in the ANB angle, the same effect could not be demonstrated in the control group. This finding further supports the fact that there is no self-correction of class II malocclusion and that functional treatment is beneficial for the patient.

Several authors underline the importance of keeping control of the vertical dimensions while correcting sagittal discrepancies [16, 20]. This is an imperative in patients with a tendency for posterior rotation of the mandible. Treatment with activator without a headgear showed effective condylar growth and change in chin position; however, these changes were not in the desired sagittal direction, rather in the vertical one [21]. In the present study, the effects of the two appliances on vertical measurements are similar; still, the AH appliance seemed to have some tendency to control the vertical dimension by promoting anterior rotation of the mandible and this is a consistent finding [7, 9, 19, 22]. Posttreatment changes in mandibular plane inclination were not observed in the TB group. This is in accordance with most studies; however, an increased mandibular plane inclination has also been reported [23, 24]. It should be emphasized that individual growth pattern varies and must be seen as an important factor contributing to the divergent treatment response.

Dentoalveolar changes played a dominant role in class II malocclusion correction in both groups, which is in agreement with other reports [11, 13]. Retroclination of maxillary incisors is a consistent finding in many other TB [2, 6, 8] and AH studies [9, 16, 19]. A more pronounced retrusion of the upper incisors was found in the AH group, which may reflect the additional headgear forces acting posteriorly on the maxillary apical base and alveolar structures. Retroclination and retrusion of prominent maxillary incisors may have a preventive effect since large overjet doubles the risk of dental trauma [25]. The most prominent dentoalveolar effect in the TB group was proclination and protrusion of the mandibular incisors compared to the AH group. Changes in the inclination of the lower incisors in functional appliances studies are contradictory and probably not sufficiently controlled by their capping with acrylic [8, 13, 18, 22].

At the end of the treatment, both treatment groups showed similar reduction of the profile convexity and retrusion of the upper lip. These results are in agreement with previous studies [9, 16, 24, 26, 27]. However, retrusion of the lip relative to the nose-chin line may reflect growth of the nose but also more forward chin position induced by functional treatment. It should also be noted that there is a large variation in treatment response for most of the soft-tissue parameters and sometimes the magnitude of the changes may not be perceived as clinically significant [28].

The discriminant analysis revealed that there was a greater difference between the control group and the two treated groups than that between the TB and AH group. The majority of the changes could be attributed to treatment with either of the two appliances, but the treatment effect was more dentoalveolar than skeletal compared to the controls.

## 5. Conclusions

Both AH and TB appliances contributed successfully to the correction of class II division 1 malocclusion when compared to the untreated growing class II subjects producing predominantly dentoalveolar effects. TB appliance leads to more pronounced protrusion and proclination of the mandibular incisors than the AH group. Treatment with TB results in some supplementary mandibular length growth while AH exerted some tendency to more control of the vertical dimension of the lower anterior facial height. Normal growth pattern in untreated class II subjects comprises forward and downward growth displacement of the maxilla and the mandible without major changes in basal sagittal relation between the jaws. Clinical relevance of these findings is that early treatment may correct or at least ameliorate class II division I malocclusion which is not self-corrective.

## Figures and Tables

**Table 1:** Cephalometric variables used in the study.

Number	Variable	Unit	Description
1	NSBa	°	Cranial base angle expression of the flexion of the cranial base (nasion-sella-basion)
2	SNA	°	Angle of anterior part of cranial base (S-N) and point A (subspinale) on maxilla
3	SNB	°	Angle of anterior part of cranial base (S-N) and point B (supramentale) on mandible
4	ANB	°	Angle between point nasion and point on maxilla and mandible, basal sagittal relation between the jaws
5	A-NPg	mm	The shortest distance from A point to the facial plane (N-Pg), expression of the skeletal convexity of the face
6	NL/NSL	°	Angle between the nasal line (anterior to posterior nasal spine ANS-PNS) and the nasion-sella line, expression of the tilting of the maxilla relative to the anterior cranial base
7	MP/NSL	°	Angle of mandibular plane (Go-Me) relative to the anterior cranial base
8	MP/NL	°	Angle of mandibular plane (Go-Me) and nasal line
9	FA/NBa	°	Lower angle between the facial axis (pterygoid point-gnathion) and the nasion-basion line, expression of the growth direction of the chin and the relationship, facial height and depth
10	UFH	mm	Upper facial height (middle third of the face), distance from nasion point to spina nasalis anterior measured perpendicular to Frankfort horizontal (FH)
11	LFH	mm	Lower facial height (lower third of the face), distance from spina nasalis anterior to menton measured perpendicular to FH
12	UFH/LFH	%	Upper to lower facial height ratio
13	Co-A	mm	Distance from condylion to A point; measurement of the effective length of midface
14	Co-Gn	mm	Distance from condylion to gnathion; measurement of the effective length of mandible
15	Max Mand diff	mm	Maxillomandibular differential length, the difference between the effective mandibular length (Co-Gn) and the effective midface length (Co-A): gives an indication of the sagittal discrepancy between maxilla and mandible
16	−1/MP	°	Superoposterior angle of lower incisor long axis and mandibular plane
17	+1/NSL	°	Inferoposterior angle of upper incisor long axis and a nasion-sella line
18	−1/A-Pg angle	°	Angle between the long axis of the lower incisor and A-Pg line
19	+1/A-Pg angle	°	Angle between the long axis of the upper incisor and A-Pg line
20	−1/A-Pg distance	mm	Distance from the midpoint of the incisal edge of the most prominent mandibular incisor to A-Pg line, expression of the protrusion of the lower incisors
21	+1/A-Pg distance	mm	Distance from the midpoint of the incisal edge of the most prominent maxillary incisor to A-Pg line, expression of the protrusion of the upper incisors
22	Gl′-Sn-Pg′	°	Lower angle formed by the line from glabella to subnasale and the line from soft tissue pogonion to subnasale, expression of the convexity of the soft tissue profile
23	Cm-Sn-Ls	°	Nasolabial angle, expression of dentoalveolar protrusion
24	Li-E	mm	Distance from lower lip (labrale inferius) to Prn-Pg′ (Ricketts' Esthetic line)
25	Ls-E	mm	Distance from upper lip (labrale superius) to Prn-Pg′ (Ricketts' Esthetic line)
26	Li-S	mm	Distance from lower lip to Cm-Pg′ (Steiner's line)
27	Ls-S	mm	Distance from upper lip to Cm-Pg′ (Steiner's line)
28	OJ	mm	Distance between the incisal edges of the most prominent maxillary and mandibular incisors, measured parallel to the occlusal line

**Table 2:** Gender, age, and treatment duration of the Twin Block (TB), Activator headgear (AH) and untreated, control (CTRL) group.

	Treatment group			*p*
	TB	AH	CTRL	
Female gender (*N*; percentage)	15 (60)	13 (52)	28 (56)	0.850^*∗*^
Age before treatment (years)				
Median (interquartile range)	11 (10–12)	10 (9–11)	11 (10-11)	0.336^*∗∗*^
Min–max	9–13	8–12	8–13	
Mean observation period (months)				
Mean ± std. deviation	14.2 ± 4.8	15.4 ± 5.5	14.9 ± 5.2	0.745^*∗∗∗*^
Min–max	8–24	12–24	10–24	

^*∗*^ *χ* ^2^ test. ^*∗∗*^Kruskal-Wallis test. ^*∗∗∗*^ANOVA.

**Table 3:** Pretreatment (T1) and posttreatment (T2) values of the investigated variables in the Twin Block (TB) and activator-headgear (AH) groups.

	TB		*p* ^*∗*^	*r* ^*∗∗*^	AH		*p* ^*∗*^	*r* ^*∗∗*^
	T1	T2			T1	T2		
	Mean ± SD	Mean ± SD			Mean ± SD	Mean ± SD		
NSBa	131.0 ± 4.5	130.9 ± 4.6	0.752	0.065	128.3 ± 5.1	127.4 ± 5.0	**0.014**	0.476
SNA	80.1 ± 3.2	79.8 ± 3.6	0.118	0.315	81.8 ± 2.8	81.7 ± 2.7	0.432	0.161
SNB	74.2 ± 3.0	75.3 ± 3.3	**<0.001**	0.751	76.2 ± 2.5	77.4 ± 2.5	**<0.001**	0.835
ANB	5.9 ± 1.6	4.4 ± 1.7	**<0.001**	0.751	5.7 ± 1.7	4.2 ± 1.7	**<0.001**	0.899
A-NPg	4.9 ± 2.1	3.7 ± 2.3	**<0.001**	0.661	4.2 ± 2.2	2.9 ± 2.4	**<0.001**	0.831
NL/NSL	7.9 ± 2.9	7.6 ± 3.1	0.359	0.187	5.4 ± 2.5	5.2 ± 2.6	0.420	0.165
MP/NSL	35.2 ± 4.7	35.3 ± 5.2	0.925	0.019	33.3 ± 5.0	32.4 ± 4.7	**0.011**	0.488
MP/NL	27.3 ± 4.5	27.7 ± 4.8	0.431	0.162	27.9 ± 4.6	27.2 ± 4.6	0.086	0.343
FA/NBa	86.1 ± 3.4	86.3 ± 3.7	0.614	0.104	87.5 ± 3.7	87.8 ± 4.0	0.299	0.212
UFH	47.7 ± 3.0	49.3 ± 3.4	**0.001**	0.597	46.2 ± 4.8	46.7 ± 3.3	0.534	0.128
LFH	56.8 ± 4.5	59.9 ± 4.7	**<0.001**	0.784	57.3 ± 5.7	58.8 ± 4.4	0.154	0.288
UFH/LFH	84.4 ± 8.0	82.8 ± 7.2	**0.014**	0.474	80.7 ± 4.9	79.6 ± 5.3	0.157	0.286
Co-A	81.0 ± 5.3	83.0 ± 4.3	**0.016**	0.467	83.0 ± 8.2	82.6 ± 4.2	0.799	0.053
Co-Gn	99.9 ± 6.1	105.0 ± 6.3	**<0.001**	0.768	102.6 ± 10.4	105.1 ± 6.0	0.162	0.283
Max Mand diff	18.9 ± 3.1	22.0 ± 4.3	**<0.001**	0.743	19.6 ± 3.6	22.4 ± 3.5	**<0.001**	0.784
−1/MP	97.7 ± 7.4	100.7 ± 7.4	**0.002**	0.572	95.5 ± 7.6	96.1 ± 6.4	0.456	0.153
+1/NSL	107.6 ± 7.1	100.5 ± 6.4	**<0.001**	0.803	110.1 ± 6.7	102.7 ± 6.3	**<0.001**	0.772
−1/A-Pg angle	22.0 ± 5.8	27.5 ± 5.2	**<0.001**	0.765	21.0 ± 7.3	23.7 ± 5.1	**0.002**	0.583
+1/A-Pg angle	38.5 ± 5.5	28.9 ± 4.7	**<0.001**	0.893	37.9 ± 5.6	27.5 ± 5.2	**<0.001**	0.867
−1/A-Pg distance	0.3 ± 2.0	2.6 ± 1.7	**<0.001**	0.875	0.0 ± 2.3	1.4 ± 2.0	**<0.001**	0.775
+1/A-Pg distance	9.3 ± 1.9	6.4 ± 1.9	**<0.001**	0.915	9.2 ± 2.0	5.6 ± 2.0	**<0.001**	0.861
Gl′-Sn-Pg′	20.3 ± 5.3	18.2 ± 5.6	**0.002**	0.583	18.5 ± 5.8	16.4 ± 5.2	**0.002**	0.586
Cm-Sn-Ls	116.9 ± 11.9	118.1 ± 10.2	0.589	0.111	107.7 ± 8.5	113.8 ± 11.3	**0.005**	0.533
Li-E	0.1 ± 2.5	−0.5 ± 2.5	0.120	0.313	−0.6 ± 3.5	−1.5 ± 2.8	**0.020**	0.455
Ls-E	−0.5 ± 2.0	−2.2 ± 2.0	**<0.001**	0.701	−0.1 ± 2.2	−1.9 ± 2.4	**<0.001**	0.823
Li-S	1.1 ± 2.4	0.7 ± 2.3	0.327	0.200	0.7 ± 3.3	−0.1 ± 2.8	0.050	0.389
Ls-S	1.3 ± .7	−0.2 ± 1.6	**<0.001**	0.661	2.0 ± 2.0	0.1 ± 2.2	**<0.001**	0.861
OJ	9.0 ± 2.5	3.8 ± 1.8	**<0.001**	0.956	9.2 ± 2.3	4.3 ± 1.9	**<0.001**	0.888

^*∗*^Paired samples *t*-test.

^*∗∗*^Effect size calculated by using the formula *r* = √*t*^2^/(*t*^2^ + *df*). Cohen criteria for interpretation of effect size were used: *r* = 0.1–0.3 = small effect size, 0.3–0.5 = medium, and >0.5 = large.

**Table 4:** Mean values of the investigated variables in the untreated, control group at the same pretreatment (T1) and posttreatment (T2) age as the treated groups.

	T1	T2	*p* ^*∗*^	*r* ^*∗∗*^
	Mean ± SD	Mean ± SD		
NSBa	130.9 ± 4.2	130.7 ± 4.7	0.514	0.093
SNA	82.1 ± 2.4	82.4 ± 2.5	0.174	0.193
SNB	75.8 ± 2.8	76.3 ± 2.6	**0.005**	0.384
ANB	6.3 ± 2.0	6.0 ± 1.9	0.204	0.181
A-NPg	5.2 ± 2.0	5.1 ± 2.1	0.479	0.101
NL/NSL	6.6 ± 3.3	6.7 ± 3.5	0.772	0.042
MP/NSL	35.0 ± 4.8	34.9 ± 5.0	0.625	0.070
MP/NL	28.4 ± 5.0	28.2 ± 5.3	0.526	0.091
FA/NBa	87.9 ± 4.3	88.2 ± 4.2	0.304	0.147
UFH	46.3 ± 3.1	47.6 ± 3.4	**<0.001**	0.581
LFH	56.5 ± 5.2	57.9 ± 6.0	**0.001**	0.463
UFH/LFH	82.3 ± 6.6	82.8 ± 7.3	0.535	0.089
Co-A	81.3 ± 4.9	82.7 ± 5.3	**<0.001**	0.512
Co-Gn	99.8 ± 5.9	102.4 ± 6.4	**<0.001**	0.761
Max Mand diff	18.5 ± 3.2	19.7 ± 4.0	**0.001**	0.461
−1/MP	97.8 ± 5.5	96.9 ± 5.5	0.105	0.230
+1/NSL	102.7 ± 7.2	103.1 ± 7.2	0.487	0.100
−1/A-Pg angle	22.7 ± 5.1	22.5 ± 5.2	0.708	0.054
+1/A-Pg angle	32.8 ± 6.7	32.4 ± 6.8	0.345	0.135
−1/A-Pg distance	1.3 ± 2.3	1.2 ± 2.4	0.749	0.046
+1/A-Pg distance	6.9 ± 2.2	7.1 ± 2.7	0.144	0.207
Gl′-Sn-Pg′	16.4 ± 4.4	16.8 ± 4.3	0.258	0.161
Cm-Sn-Ls	110.0 ± 13.9	112.9 ± 9.6	**0.037**	0.293
Li-E	1.4 ± 2.2	1.1 ± 2.3	0.270	0.157
Ls-E	0.4 ± 2.2	−0.1 ± 2.1	**0.017**	0.334
Li-S	2.3 ± 2.2	2.2 ± 2.3	0.783	0.039
Ls-S	2.1 ± 2.0	2.0 ± 2.2	0.829	0.031
OJ	5.6 ± 2.1	5.9 ± 2.2	0.068	0.258

^*∗*^Paired samples *t*-test.

^*∗∗*^Effect size.

**Table 5:** Comparison of the treatment changes (Δ) in the Twin Block (TB) and activator-headgear (AH) group and untreated controls (CTRL).

	ΔTB	ΔAH	ΔCTRL	*p* ^*∗*^	*η* ^2*∗∗*^
	Mean ± SD	Mean ± SD	Mean ± SD		
NSBa	−0.1 ± 2.1	−0.9 ± 1.6	−0.2 ± 2.1	0.313	0.024
SNA	−0.4 ± 1.2	−0.2 ± 1.2	0.3 ± 1.5	0.087	0.049
SNB	1.1 ± 1.0	1.2 ± 0.8	0.6 ± 1.3	**0.036**	0.066
ANB	−1.5 ± 1.3^a^	−1.4 ± 0.7^a^	−0.3 ± 1.5^b^	**<0.001**	0.179
A-NPg	−1.2 ± 1.3^a^	−1.3 ± 0.9^a^	−0.1 ± 1.4^b^	**<0.001**	0.161
NL/NSL	−0.4 ± 1.9	−0.3 ± 1.6	0.1 ± 2.1	0.588	0.011
MP/NSL	−0.0 ± 1.7	−0.9 ± 1.7	−0.1 ± 2.1	0.138	0.040
MP/NL	0.4 ± 2.4	−0.7 ± 1.9	−0.2 ± 2.6	0.283	0.026
FA/NBa	0.2 ± 2.0	0.3 ± 1.5	0.3 ± 2.0	0.976	0.001
UFH	1.6 ± 2.2	0.5 ± 4.0	1.3 ± 1.9	0.295	0.025
LFH	3.0 ± 2.4	1.5 ± 5.1	1.4 ± 2.6	0.119	0.043
UFH/LFH	−1.7 ± 3.2	−1.1 ± 3.8	0.5 ± 5.8	0.134	0.041
Co-A	2.0 ± 3.9	−0.3 ± 6.7	1.4 ± 2.4	0.114	0.044
Co-Gn	5.1 ± 4.4	2.5 ± 8.6	2.6 ± 2.2	0.086	0.049
Max Mand diff	3.1 ± 2.9^a^	2.8 ± 2.3^a^	1.1 ± 2.2^b^	**0.001**	0.126
−1/MP	3.0 ± 4.4^a^	0.5 ± 3.6^b^	−0.9 ± 3.8^b^	**<0.001**	0.145
+1/NSL	−7.0 ± 5.3^a^	−7.3 ± 6.2^a^	0.4 ± 4.0^b^	**<0.001**	0.378
−1/A-Pg angle	5.5 ± 4.8^a^	2.7 ± 3.8^b^	−0.2 ± 3.3^c^	**<0.001**	0.283
+1/A-Pg angle	−9.6 ± 4.9^a^	−10.4 ± 6.1^a^	−0.5 ± 3.4^b^	**<0.001**	0.528
−1/A-Pg distance	2.3 ± 1.3^a^	1.3 ± 1.1^b^	−0.1 ± 1.2^c^	**<0.001**	0.416
+1/A-Pg distance	−2.9 ± 1.3^a^	−3.6 ± 2.2^a^	0.2 ± 1.1^b^	**<0.001**	0.592
Gl′-Sn-Pg′	−2.1 ± 3.0^a^	−2.0 ± 2.9^a^	0.4 ± 2.3^b^	**<0.001**	0.182
Cm-Sn-Ls	1.2 ± 11.0	6.1 ± 10.0	2.9 ± 9.5	0.207	0.032
Li-E	−0.6 ± 1.8	−0.9 ± 1.9	−0.3 ± 1.6	0.290	0.025
Ls-E	−1.8 ± 1.8^a^	−1.8 ± 1.3^a^	−0.4 ± 1.2^b^	**<0.001**	0.205
Li-S	−0.4 ± 1.8	−0.8 ± 2.0	−0.1 ± 1.6	0.212	0.032
Ls-S	−1.5 ± 1.7^a^	−1.9 ± 1.1^a^	−0.0 ± 1.4^b^	**<0.001**	0.261
OJ	−5.2 ± 1.6^a^	−4.9 ± 2.6^a^	0.3 ± 1.2^b^	**<0.001**	0.712

^*∗*^ANOVA with Student-Newman-Keuls post hoc tests. Groups in the same row that share the same superscript letter do not differ significantly.

^*∗∗*^Effect size calculated according to formula: *η*^2^ = between groups sum of squares/total sum of squares. Cohen criteria for interpretation of effect size were used: *η*^2^ = 0.02–0.13 = small effect size, 0.13–0.26 = medium, and >0.26 = large.

**Table 6:** Structural matrix of canonical discriminant functions.

	Function	
	1	2
Δ+1/A-Pg distance	−0.699^*∗*^	−0.394
Δ−1/A-Pg distance	0.476^*∗*^	−0.453
Δ+1/NSL	−0.458^*∗*^	−0.108
ΔLs-E	−0.298^*∗*^	−0.076
ΔGl′-Sn-Pog′	−0.277^*∗*^	−0.025
ΔANB	−0.275^*∗*^	−0.008
ΔSNB	0.153^*∗*^	0.112
ΔSNA	−0.131^*∗*^	0.087
ΔUFH/LFH	−0.120^*∗*^	0.062
Δ−1/MP	0.210	−0.387^**∗**^
ΔCo-A	−0.034	−0.383^**∗**^
ΔMP/NSL	−0.045	−0.351^**∗**^
ΔCo-Gn	0.080	−0.340^**∗**^
ΔLi-E	−0.082	−0.150^**∗**^

^*∗*^Largest absolute correlation between each variable and any discriminant function. Variables ordered by absolute size of correlation within function.

## References

[1] Tulloch J. F., Phillips C., Proffit W. R. (1998). Benefit of early Class II treatment: progress report of a two-phase randomized clinical trial. *American Journal of Orthodontics and Dentofacial Orthopedics*.

[2] Jena A. K., Duggal R., Parkash H. (2005). Orthopedic and orthodontic effects of twin-block appliance. *Journal of Clinical Pediatric Dentistry*.

[3] Varlik S. K., Gültan A., Tümer N. (2008). Comparison of the effects of Twin Block and activator treatment on the soft tissue profile. *European Journal of Orthodontics*.

[4] O'Brien K., Macfarlane T., Wright J. (2009). Early treatment for Class II malocclusion and perceived improvements in facial profile. *American Journal of Orthodontics and Dentofacial Orthopedics*.

[5] Koretsi V., Zymperdikas V. F., Papageorgiou S. N., Papadopoulos M. A. (2015). Treatment effects of removable functional appliances in patients with Class II malocclusion: a systematic review and meta-analysis. *European Journal of Orthodontics*.

[6] Baccetti T., Franchi L., Toth L. R., McNamara J. A. (2000). Treatment timing for Twin-block therapy. *American Journal of Orthodontics and Dentofacial Orthopedics*.

[7] O'Brien K., Wright J., Conboy F. (2003). Effectiveness of early orthodontic treatment with the Twin-block appliance: a multicenter, randomized, controlled trial. Part 1: dental and skeletal effects. *American Journal of Orthodontics and Dentofacial Orthopedics*.

[8] Trenouth M. J. (2002). Proportional changes in cephalometric distances during Twin Block appliance therapy. *European Journal of Orthodontics*.

[9] Öztürk Y., Tankuter N. (1994). Class II: a comparison of activator and activator headgear combination appliances. *European Journal of Orthodontics*.

[10] Lehman R., Romuli A., Bakker V. (1988). Five-year treatment results with a headgear-activator combination. *European Journal of Orthodontics*.

[11] Jena A. K., Duggal R., Parkash H. (2006). Skeletal and dentoalveolar effects of Twin-block and bionator appliances in the treatment of Class II malocclusion: a comparative study. *American Journal of Orthodontics and Dentofacial Orthopedics*.

[12] Baccetti T., Franchi L., McNamara J. A. (2005). The Cervical Vertebral Maturation (CVM) method for the assessment of optimal treatment timing in dentofacial orthopedics. *Seminars in Orthodontics*.

[13] Lerstøl M., Torget Ø., Vandevska-Radunovic V. (2010). Long-term stability of dentoalveolar and skeletal changes after activator-headgear treatment. *European Journal of Orthodontics*.

[14] Gill D. S., Lee R. T. (2005). Prospective clinical trial comparing the effects of conventional Twin-block and mini-block appliances: part 1. Hard tissue changes. *American Journal of Orthodontics and Dentofacial Orthopedics*.

[15] Dahlberg G. (1940). *Statistical Methods for Medical and Biological Students*.

[16] Marşan G. (2007). Effects of activator and high-pull headgear combination therapy: skeletal, dentoalveolar, and soft tissue profile changes. *European Journal of Orthodontics*.

[17] Ehsani S., Nebbe B., Normando D., Lagravere M. O., Flores-Mir C. (2014). Short-term treatment effects produced by the Twin-block appliance: a systematic review and meta-analysis. *European Journal of Orthodontics*.

[18] Perinetti G., Primožič J., Franchi L., Contardo L. (2015). Treatment effects of removable functional appliances in pre-pubertal and pubertal Class II patients: a systematic review and meta-analysis of controlled studies. *PLoS ONE*.

[19] Dermaut L. R., van den Eynde F., de Pauw G. (1992). Skeletal and dento-alveolar changes as a result of headgear activator therapy related to different vertical growth patterns. *European Journal of Orthodontics*.

[20] Pancherz H. (1984). A cephalometric analysis of skeletal and dental changes contributing to Class II correction in activator treatment. *American Journal of Orthodontics*.

[21] Ruf S., Baltromejus S., Pancherz H. (2001). Effective condylar growth and chin position changes in activator treatment: a cephalometric roentgenographic study. *The Angle Orthodontist*.

[22] Sidlauskas A. (2005). The effects of the Twin-block appliance treatment on the skeletal and dentolaveolar changes in Class II Division 1 malocclusion. *Medicina (Kaunas)*.

[23] Toth L. R., McNamara J. A. (1999). Treatment effects produced by the twin-block appliance and the FR-2 appliance of Fränkel compared with an untreated Class II sample. *American Journal of Orthodontics and Dentofacial Orthopedics*.

[24] Tümer N., Gültan A. S. (1999). Comparison of the effects of monoblock and twin-block appliances on the skeletal and dentoalveolar structures. *American Journal of Orthodontics and Dentofacial Orthopedics*.

[25] Petti S. (2015). Over two hundred million injuries to anterior teeth attributable to large overjet: a meta-analysis. *Dental Traumatology*.

[26] Sharma A. A., Lee R. T. (2005). Prospective clinical trial comparing the effects of conventional Twin-block and mini-block appliances: part 2. Soft tissue changes. *American Journal of Orthodontics and Dentofacial Orthopedics*.

[27] McDonagh S., Moss J. P., Goodwin P., Lee R. T. (2001). A prospective optical surface scanning and cephalometric assessment of the effect of functional appliances on the soft tissues. *European Journal of Orthodontics*.

[28] Flores-Mir C., Major P. W. (2006). Cephalometric facial soft tissue changes with the twin block appliance in class II division 1 malocclusion patients. A systematic review. *The Angle Orthodontist*.

